# Pemetrexed in Previously Treated Non-small Cell Lung Cancer Patients with Poor Performance Status

**DOI:** 10.3779/j.issn.1009-3419.2011.01.07

**Published:** 2011-01-20

**Authors:** Young JUNG Sun, Jin YOO Su, Young SHIN Ji, Won PARK Ji, Eun LEE Jeong, Sun PARK Hee, Ock KIM Ju, Young KIM Sun

**Affiliations:** Department of Internal Medicine, Chungnam National University Hospital, Daejon, Korea

**Keywords:** Pemetrexed, Lung neoplasms, Poor performance

## Abstract

**Background and objective:**

Pemetrexed have been approved for the treatment of patients affected by advanced non-small cell lung cancner (NSCLC) in progression after first-line chemotherapy. We evaluated the activity and feasibility of pemetrexed in previously treated NSCLC.

**Methods:**

Patients with histologically or cytologically confirmed NSCLC were evaluated from April 2007 to March 2009. The patients had relapsed or progressed after prior chemotherapy treatment. Pemetrexed (500 mg/m^2^) was administered intravenously once every 3 weeks after progression to prior chemotherapy. The tumor response was evaluated according to RECIST criteria by chest CT at every 2 cycles of chemotherapy.

**Results:**

A total 61 patients were eligible for analysis. Performance status of them (100%) was over 2. The response rate and disease control rate were 14.7% and 37.7% respectively. Non-squamous cell carcinoma histology was significantly associated with a superior response rate (*P*=0.045) and disease control rate (*P*=0.008). The median survival time and the median progression free survival (PFS) time were 6.11 months and 2.17 months, respectively. Comparing the efficacy of pemetrexed in these two settings [second-line versus (12/61) more than third (49/61)], there was no significant difference in regard to median survival (11.18 months *vs* 11.46 months, *P*=0.922, 5), but PFS was more longer in third- or further-line groups than second-line group (1.39 months *vs* 2.25 months, *P*=0.015, 3).

**Conclusion:**

Pemetrexed is a feasible regimen in previously treated NSCLC with poor performance status.

## Introduction

Lung cancer is the leading cause of cancer-related deaths in Korea and worldwide^[1, 2]^. More than 80% of patients with lung cancer have non-small cell lung cancer (NSCLC) histology^[3]^. Additionally, most NSCLC patients are diagnosed with locally advanced or metastatic disease. For advanced disease, systemic chemotherapy is the standard therapy. The standard first-line therapy for patients with NSCLC is platinum-based doublet combination chemotherapy, which offers a modest survival advantage^[4]^. As second-line chemotherapeutic agents, various drugs such as docetaxel, pemetrexed, and the epidermal growth factor receptor (EGFR) tyrosine kinase inhibitors (TKIs) erlotinib and gefitinib have been used. Among them, pemetrexed is a multi-target antifolate second-line chemotherapeutic agent^[5]^ that shows effects comparable to docetaxel.

Presently, numerous NSCLC patients receive higher than third-line chemotherapy; however, a guideline for chemotherapy has not been established for these patients. Pemetrexed is less toxic than other therapeutics, and thus could be used safely in the elderly and in cases with poor Eastern Cooperative Oncology Group performance status (ECOG PS)^[7, 8]^. Thus, pemetrexed could be used for later than third-line chemotherapy. However, until now, few studies have investigated the efficacy of pemetrexed in later than third-line chemotherapy with poor PS.

In the present study, the efficacy of pemetrexed in later than third-line chemotherapy in NSCLC patients with poor PS was examined retrospectively.

## Patients and methods

### Patients

The study population consisted of 56 patients who received pemetrexed because of progression after treatment at Chungnam National University Hospital between April 2007 and March 2009. Clinicopathologic data and follow-up information were retrieved from patient medical records. All patients had histological or cytological confirmation of locally advanced or metastatic NSCLC, stage Ⅲb or Ⅳ [tumor node metastasis (TNM) staging], and poor ECOG PS (≥2).

### Treatment

All patients received 500 mg/m^2^ pemetrexed as a 10 min intravenous infusion on day 1 and every 21 days thereafter. Folic acid supplementation (1, 000 mg) was orally administered daily beginning approximately 1-2 weeks before the first dose of pemetrexed and continued until 3 weeks after treatment discontinuation. A 1, 000 mg vitamin B_12_ injection was administered intramuscularly approximately 1-2 weeks before the first dose of pemetrexed and was repeated approximately every 9 weeks until 3 weeks after therapy discontinuation. Dexamethasone was administered twice daily on the day before, the day of and the day after each dose of pemetrexed. Treatment was continued until disease progression, unacceptable toxicity, or the patient or physician decided to discontinue therapy.

### Response

Tumor responses were assessed with computed tomography every two or three cycles to evaluate treatment responses for documentation of disease progression. Additionally, for cases with headache, nausea, vomiting, and other neurological symptoms (which together could indicate brain metastasis), systemic metastasis was evaluated by performing brain magnetic resonance imaging. For cases suspected to have bone metastasis, systemic metastasis was evaluated by bone scan or positron emission tomography-CT. Objective tumor responses were based on Response Evaluation Criteria in Solid Tumors (RECIST) criteria^[9]^.

### Statistical analysis

Statistical analysis was performed using the Statistical Package for the Social Sciences (SPSS) software (version 17.0; SPSS, Inc., Chicago, IL, USA). The survival period was from the day of the first administration of pemetrexed to death or the date that the follow-up observation was terminated. The progression-free survival (PFS) encompassed the time from the first cycle of pemetrexed therapy to documented progression or death from any cause until the date of the last follow-up visit for patients who were still alive and whose cancer had not progressed. For the analysis of survival period and PFS, the *Kaplan-Meier* method was used, and *P*-values were obtained based on the *Log-rank* test.

For comparison of the characteristics of the patient groups, treatment response rate, and control of disease, the chi-square test was used. For analysis of factors mediating effects on survival, the Logistic regression model was used. Statistical significance was deemed to be *P* < 0.05.

## Results

### Patient characteristics and distribution

Among 56 patients, 11 (19.6%) were treated with pemetrexed as second-line therapy, and 45 (80.4%) were administered pemetrexed as later than third-line therapy. The study group included 38 males (67.9%) and 18 females (32.1%). The age distribution was 43-81 years: 15 patients (26.8%) were > 70 years, and 41 patients (73.2%) were < 70 years. For patients > 70 years, 63.6% were in secondline treatment, whereas 17.8% were in later than third-line treatment. All 56 patients had an ECOG PS ≥2. In total, 32 patients had non-squamous cell carcinoma (adenocarcinoma, *n*=29; indeterminate non-small-cell carcinoma, *n*=3), and 24 (42.9%) patients had squamous cell carcinoma. Regarding disease stage, 16 patients exhibited less than stage Ⅲb, and 40 exhibited stage Ⅳ disease. Almost all patients had been treated with a platinum-based chemotherapy as first-line treatment: 38 underwent a gemcitabine-platinum regimen, 10 received a taxane-platinum regimen, and 5 were treated with an irinotecan-platinum regimen. The best response to first-line therapy was as follows: partial response (PR) in 25 patients (44.6% of evaluable patients), stable disease (SD) in 15 patients (26.8%), and progressive disease (PD) in 16 patients (28.6%) (Tab 1).

**1:** Patients characteristics (*n*=56)

Characteristic	2^nd^ line [*n* (%)]	3^rd^ or further line [*n* (%)]	Total
All patients	11 (100%)	45 (100%)	56
Age (years)			
≥70	7 (63.6%)	8(17.8%)	15
< 70	4 (36.4%)	37 (82.2%)	41
Gender			
Male	8 (72.7%)	30 (66.7%)	38
Female	3 (27.3%)	15 (33.3%)	18
ECOG PS			
2	7 (63.6%)	32 (71.1%)	39
≥3	4 (36.4%)	13 (28.9%)	17
Histology			
Adenocarcinoma	3 (27.3%)	26 (57.8%)	29
Squamous cell carcinoma	6 (54.5%)	18 (40.0%)	24
NSCLC	2 (18.2%)	1 (2.2%)	3
Stage			
≤Ⅲb	3 (27.3%)	13 (28.9%)	16
Ⅳ	8 (72.7%)	32 (71.1%)	40
First-line therapy			
Gemcitabine-platinum	6 (54.5%)	32 (71.2%)	38
Taxane-platinum	1 (9.1%)	9 (20.0%)	10
Irinotecan-platinum	3 (27.3%)	2 (4.4%)	5
Others	1 (9.1%)	2 (4.4%)	3
Best response to first-line therapy			
PR-CR	4 (36.4%)	21 (46.7%)	25
SD	2 (18.2%)	13 (28.9%)	15
PD	5 (45.5%)	11 (24.4%)	16
Metastasis			
No metastasis	3 (27.3%)	13 (28.9%)	16
Brain	1 (9.1%)	4 (8.9%)	5
Bone	0	2 (4.4%)	2
Adrenal gland	2 (18.2%)	4 (8.9%)	6
Lung to lung	1 (9.1%)	10(22.2%)	11
Others	0	5(11.1%)	5
Multiple organs	4 (36.4%)	7 (15.6%)	11
NSCLC: non-small cell lung cancer; PR: partial response; CR: complete response; SD: stable disease; PD: progressive disease.			

### Response

Among 56 cases, complete remission was achieved in one case (1.8%), partial remission was achieved in eight cases (14.3%), no change occurred in 14 cases (25.0%), and progressive lesions were found in 33 cases (58.9%). The treatment response rate was 16.1%, and the disease control rate was 41.1% (Tab 2). The median survival was 6.11 months, and the median PFS was 2.17 months.

**2:** Best response data for each line pemetrexed therapy

Best response	2^nd^ line (*n*=11)	3^rd^ line (*n*=13)	4^th^ line (*n*=20)	5^th^ line or further (*n*=12)	Total (*n*=56)
Complete response	0	1 (7.7%)	0	0	1 (1.8%)
Partial response	0	3 (23.1%)	3 (15.0%)	2 (16.7%)	8 (14.3%)
Stable disease	3 (27.3%)	3 (23.1%)	6 (30.0%)	2 (16.7%)	14 (25.0%)
Progressive disease	8(72.7%)	6 (46.2%)	11 (55.0%)	8 (66.7%)	33 (58.9%)
Disease control	3 (27.3%)	7 (53.8%)	9 (45.0%)	4 (33.3%)	23 (41.1%)

### Subgroup analysis

The non-squamous cell cancer group included 18 cases (56.2%), and in comparison with the 5 cases in the squamous cell cancer group (20.8%), the disease control rate in the former group was significantly higher (*P*=0.018). Similarly, the treatment response rate for the non-squamous epithelial cancer group was 25.0% (8 cases), which was significantly higher than the 4.1% (1 case) for the squamous epithelial cancer group (*P*=0.045). By multivariate analysis, only histology showed persistent predictive relevance (Tab 3).

**3:** Univariate and multivariate analyses for progression-free survival

Characteristic	PFS (months)	Univariate analysis	Multivariate analysis
		*P*	Odds ratio (95%CI)
All patients	3.60		
Age		0.187	0.315 (0.057-1.751)
≥70 years	3.10		
< 70 years	3.79		
Gender		0.300	2.111 (0.511-8.664)
Male	3.12		
Female	4.62		
Perfomance status		0.059	0.237 (0.053-1.058)
2	4.21		
≥3	2.22		
Histology		0.018	0.178 (0.043-0.742)
Non-squamous cell carcinoma	4.48		
Squamous cell carcinoma	2.44		
Best response to first-line therapy		0.242	0.387 (0.79-1.899)
Disease control (PR/SD)	4.02		
PD	2.58		
No. of prior systemic therapy		0.904	0.897 (0.152-5.291)
1	2.13		
≥2	3.96		

### Efficacy of third- or further-line pemetrexed therapy

Pemetrexed was administered as part of second-line (19.6% of the patients), third-line (23.2% of the patients), fourth-line (35.7% of patients), and later than fifth-line chemotherapy (21.4% of the patients). In 80.4% of patients, pemetrexed was administered as later than third-line chemotherapy. When second-line was compared with later than third-line therapy, the median PFS was higher in later than third-line therapy (1.50 months *vs* 2.25 months; *P*=0.017, 1). The median survival of second-line chemotherapy was not significantly different from that in later than third-line chemotherapy (11.18 months *vs* 11.46 months; *P*=0.922, 5).

### Toxicity

The toxicity profiles of all patients are summarized in Tab 4. Anemia was the most frequent adverse event (28.4%), followed by nausea (23%). Other adverse events were less frequent. Grade 3-4 hematologic toxicity occurred in 8.8% of cases. None of the patients required dose modifications due to toxicity.

**4:** Toxicity profiles

	Grade 1 [*n* (%)]	Grade 2 [*n* (%)]	Grade 3 [*n* (%)]	Grade 4 [*n* (%)]
Hematologic toxicity				
Neutropenia	0	2 (3.5%)	0	2 (3.5%)
Anemia	6 (10.7%)	8 (14.2%)	2 (3.5%)	
Thrombocytopenia	1 (1.7%)	3 (5.3%)	0	
Non-hematologic toxicity				
Nausea	0	8 (14.2%)	3 (5.3%)	2 (3.5%)
Diarrhea	2 (3.5%)	2 (3.5%)	0	
Constipation	0	2 (3.5%)	0	
Rash	0	2 (3.5%)	1 (1.7%)	

## Discussion

Presently, first-line chemotherapy for progressive NSCLC consists of combination platinum-based chemotherapies^[10]^. For cases with disease recurrence or progressive disease after first-line therapy, docetaxel, pemetrexed, or an EGFRTKI (erlotinib or gefitinib) may be used. Among them, the median survival of docetaxel has been shown to be significantly longer than optimal palliative therapy, and thus a survival benefit has been confirmed^[11]^.

Compared with docetaxel, pemetrexed has demonstrated a similar median survival, and thus its efficacy is accepted. Importantly, its side effects were revealed to be safer than those from docetaxel^[6, 12]^.

In our study, pemetrexed was used as later than secondline chemotherapy for NSCLC treatment. The treatment response rate was 16.1%, and the disease control rate was 41.1%. The median survival was 6.11 months, and the median PFS was 2.17 months. Compared with the squamous cell cancer group, the non-squamous cell cancer group showed a statistically significantly higher treatment response rate and disease control rate. When second-line chemotherapy was compared with third-line or later chemotherapy, the median PFS of third-line or later chemotherapy was 2.25 months, which was statistically significantly longer than that with second-line therapy; however, the medial survival was not significantly different. A total of 11 patients were treated with pemetrexed as second-line therapy. Compared to 45 patients who received it as further-line therapy, the 11 patients who received second-line therapy were older and more frequently had squamous cell carcinoma (54.5% *vs* 40%). We believe that the longer PFS in the further-line pemetrexed group is explained by these differences in age and cancer type. In our study, only histology retained significance in the multivariate analysis. Grade 3-4 hematologic toxicity occurred in 8.8% of cases. Although the present study included a large proportion of elderly and poor PS patients, the toxicity profiles were mild and acceptable compared with the JMEI trial (a pemetrexed *vs* docetaxel trial)^[6]^.

In a phase Ⅲ clinical study examining the efficacy and safety of docetaxel and pemetrexed, prognostic factors included a PS of 0-1, stage Ⅲ disease, and a relapse of > 3 months after the last chemotherapy^[6]^. In the study conducted in Korea and reported by Lee *et al*^[13]^, which assessed second-line chemotherapy for progressive NSCLC, pemetrexed monotherapy was administered for progressive NSCLC. The treatment response rate was 5.1%, and the disease control rate was 46.2%. The median survival was 7.8 months, and the median PFS was 3.1 months. Factors mediating the effects on the treatment response rate were not determined, and independent factors mediating the effects on the disease control rate were non-smoker status and adenocarcinoma cancer type.

In another study reported by Lee *et al*^[14]^, examining cases that used pemetrexed as second-line or later chemotherapy, the treatment response rate was 11%, and the disease control rate was 66%. The median survival was 13 months, and the median PFS was 2.3 months. In comparison with the squamous epithelial cancer group, the treatment response and disease control rates of the non-squamous epithelial cancer group were shown to be significantly higher, which was similar to our study.

In the study reported by Sun *et al*^[15]^, the patients receiving pemetrexed were divided into two groups: the second-line group and the third-line or later group. The percentages in these two groups were 30% and 70%, respectively, which is comparable to our study, in which the ratio of third-line or later pemetrexed patients was 80.4%. The treatment response rate was 12%, and the disease control rate was 55%. The median survival in that study was 12.8 months, and the median PFS was 3.03 months. When the PFS for second-line chemotherapy was compared with that for with third-line chemotherapy, no significant difference was detected. The only independent factor that showed a survival benefit was PS.

Longer median PFS was found in the study reported by Lee, Sun, *et al*^[14, 15]^ and this was thought to be related to the fact that 79% and 59% of subjects were in PS levels 0 and 1, respectively, whereas in our study, 100% of cases were at PS levels 2 and higher. Thus, in the studies described above, as well as in our study, for NSCLC patients with poor PS, particularly for those with non-squamous cell cancer, pemetrexed is effective as second-line or later chemotherapy. In cases when it is used as later than third-line chemotherapy, its efficacy was comparable to that for second-line therapy, and as in our study, for cases whose ECOG PS is > 2, it was found to be efficacious.
